# The association between bowel resection and the risk of nontyphoidal salmonella infection: a nationwide propensity score-matched cohort study

**DOI:** 10.1038/s41598-021-81224-5

**Published:** 2021-01-14

**Authors:** Kuang-Tsu Yang, Sin-Ei Juang, Yao-Min Hung, James Cheng-Chung Wei, Hei-Tung Yip, Renin Chang

**Affiliations:** 1 Division of Gastroenterology and Hepatology, Department of Internal Medicine, Kaohsiung Veterans General Hospital, Kaohsiung, Taiwan (R.O.C.); 2 Department of Anesthesiology, Kaohsiung Chang Gung Memorial Hospital, Kaohsiung, Taiwan (R.O.C.); 3 Department of Internal Medicine, Kaohsiung Municipal United Hospital, Kaohsiung, Taiwan (R.O.C.); 4 School of Medicine, National Yang-Ming University, Taipei, Taiwan (R.O.C.); 5 Yuh-Ing Junior College of Health Care and Management, Kaohsiung, Taiwan (R.O.C.); 6 Graduate Institute of Integrated Medicine, China Medical University, Taichung, Taiwan (R.O.C.); 7 Division of Allergy, Immunology and Rheumatology, and Institute of Medicine, Chung Shan Medical University Hospital, Taichung, Taiwan (R.O.C.); 8 Management Office for Health Data, China Medical University Hospital, Taichung, Taiwan (R.O.C.); 9 Department of Emergency Medicine, Kaohsiung Veterans General Hospital, No. 386, Dazhong 1st Rd., Zuoying Dist., Kaohsiung City, 813414 Taiwan (R.O.C.); 10 Department of Recreation Sports Management, Tajen University, Pingtung, Taiwan (R.O.C.)

**Keywords:** Microbiology, Gastroenterology

## Abstract

Nontyphoidal salmonella (NTS) infection has a high mortality rate. Bowel resections affect gut microbiota and immune function, and the association between bowel resection and NTS infection in human beings has not been addressed. We conducted a nationwide propensity score (PS)-matched cohort study to clarify this association. Data from the Longitudinal Health Insurance Database of Taiwan were used to establish a case-cohort with bowel resections from 2000 to 2013. Informed consent was waived by the Institutional Review Board of China Medical University Hospital (CMUH104-REC2-115) because all personal identifying information used had been de-identified. Each case was matched with one control without any bowel resection according to age, gender, index date, and propensity score (PS). Cumulative incidences of and hazard ratios (HRs) for NTS infection development were analyzed. The incidence of NTS infection was greater in patients with a bowel resection than in the control group (2.97 vs. 1.92 per 10,000 person-years), with an adjusted hazard ratio (aHR) of 1.64 (95% CI = 1.08–2.48). The incidence of NTS infection increased significantly for cases with small bowel resections and right hemicolectomies. Age (31–40 and > 50 years), hypertension, chronic kidney disease, chronic obstructive pulmonary disease, and autoimmune diseases were significant risk factors of NTS infection. Stratification analysis revealed that patients without comorbidities were prone to NTS infection after bowel resections. The increased risk of developing NTS infection could be related to the bowel resection. Specific age groups and comorbidities also contribute to increased risk of NTS infection.

## Introduction

Nontyphoidal salmonella (NTS) infection is an important issue. The incidence of invasive NTS was 7.5 cases per 100,000 people worldwide in 2017^1^. The main risk factors of hosts include extreme age, immunocompromised status and immunomodulator use^2,3^. NTS bacteremia usually results in higher rate of mortality in patients with underlying diseases^4^.

Our gut mucosal immune system can keep pathobionts in check, restrict microbial overgrowth, and react to invading microorganisms that breach the intestinal chemical and physical barriers^5^. Several bacteria have some controlling effects on *S. enterica*, such as *Bifidobacterium thermophilum* and *Lactobacillus casei*^6,7^. Argüello et al. discovered that manipulating certain taxa of microbiota could decrease salmonella infection for pigs^8^.

In human beings, bowel resection strongly influences gut microbiota and intestinal healing^9^. In the small intestine, the main reported bacterial species included Bacilli (Firmicutes), Streptococcaceae (Firmicutes), Actinobacteria, Clostridium and Bacteroides^5^. Phyla Bacteroidetes and Firmicutes can dominate the mucosa-associated bacteria from the distal small intestine and the colon^5^. Specialized bacteria (e.g., Clostridium, Lactobacillus, or Enterococcus) able to adhere to mucus (as a nutrient source) and different species in feces (belonging to Bacteroides, Bifidobacterium, Streptococcus, Enterobacteriaceae, Enterococcus, Clostridium, Lactobacillus, and Ruminococcus) were identified^5^. Kunz et al. indicated that gastrectomy could lead to *S. enteritis*. This might result from relative or absolute achlorhydria, decreased hydrogen ion concentration, rapid emptying of food into the small intestine and the colon and altered bacterial flora^10^.

However, currently the association between bowel resection (except the stomach) and NTS infection was still not clarified. The present nationwide propensity score (PS)-matched cohort study was carried out in Taiwan to evaluate the risk of NTS infection after bowel resection.

## Materials and methods

### Data source and ethics statement

We extracted the claims-based data from the Longitudinal Health Insurance Database 2000 (LHID), which is a dataset included in Taiwan’s National Health Insurance Research Database (NHIRD). The NHIRD contains patients’ characteristics, medical information, total expenditure, and diagnoses coded in the International Classification of Diseases, Ninth Revision, Clinical Modification (ICD-9-CM) format. Taiwan’s National Health Insurance (NHI) programme is administered by the government and covers more than 99% of the 23.4 million population of Taiwan^11^. Data for the LHID were collected by systematically and randomly sampling from the NHIRD; this database includes the data of one million individuals. Information on demographic data, inpatient and outpatient cares, date of clinic visit or hospitalization, and prescriptions were available in the database for the period from 1997 to 2013. The NHRI reported no significant differences between the patients in the LHID and those in the original NHIRD^12^. The studies involving human participants were reviewed and approved by the Institutional Review Board of China Medical University Hospital (approval number CMUH104-REC2-115). Written informed consent for participation was not required for this study in accordance with national legislation and institutional requirements. Our research adhered strictly in accordance with relevant guidelines/regulations.

### Study subjects and design

**Figure 1:**
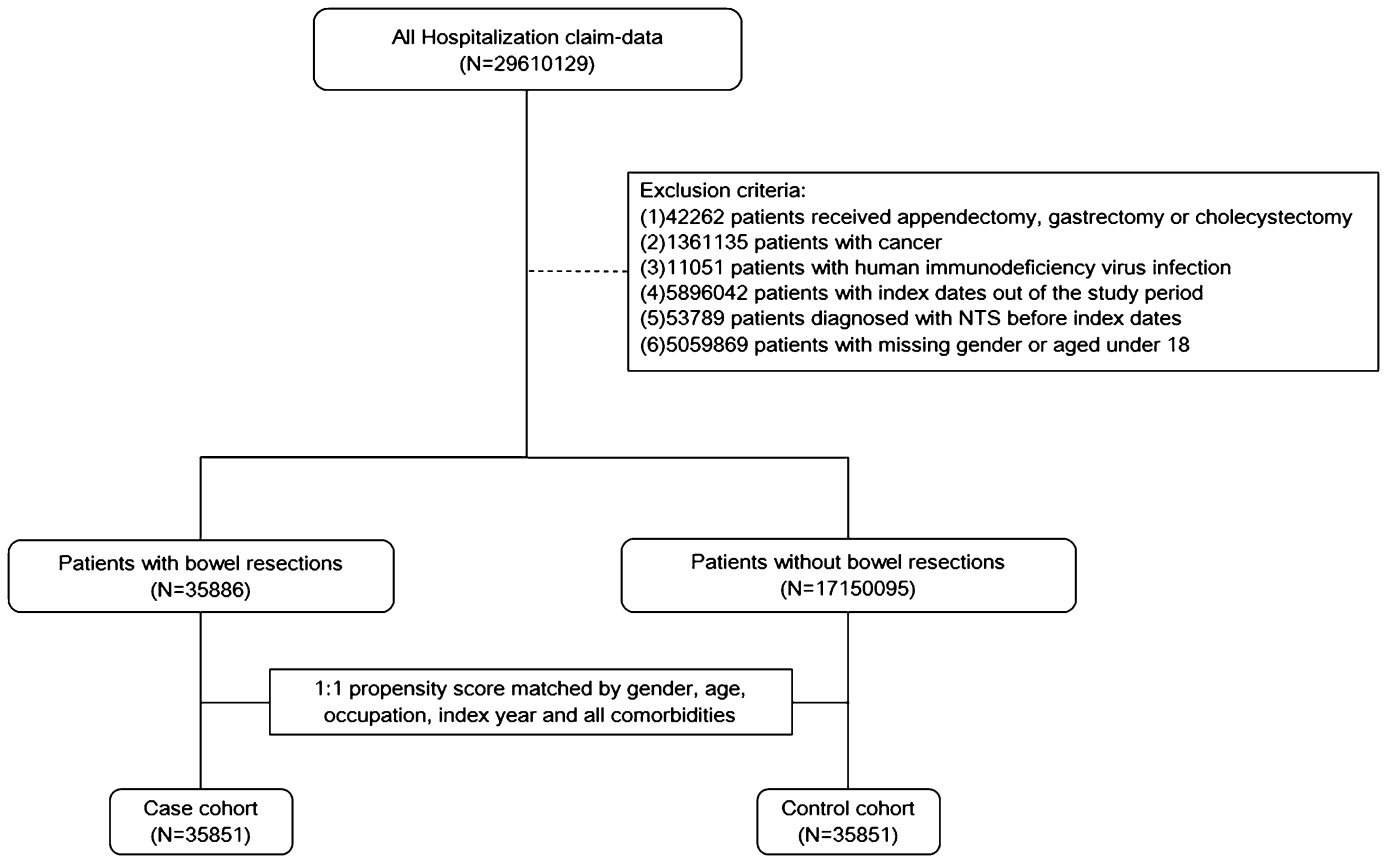
Study flowchart.

A bowel resection-related case group was established, including patients aged 18 years and over who had received a bowel resection (small bowel, large bowel, or both) from 2000 to 2013. We included the cases identified by the procedure codes of 45.6 (small bowel resection), 45.73 (right hemicolectomy), 45.74 (transverse colon resection), 45.75 (left hemicolectomy), 45.76 (sigmoidectomy), 45.8 (total colectomy), or 45.9 (bowel anastomosis). We defined the index date as the operation date. We excluded patients with appendectomy/gastrectomy/cholecystectomy, any kind of cancer, human immunodeficiency virus infection, index dates out of the study period, diagnosis of NTS, or missing gender or aged under 18 before index dates (Fig. 1). The outcome was the development of NTS (ICD-9-CM codes: 003.xx) after the index date.

### Propensity score matching and covariates

A matched control was assigned to each case (Fig. 1). We used the PS-matching method to account for baseline differences^13^. The observed covariates in the logistic regression model to generate the PS were hypertension (ICD9 codes: 401–405), diabetes (ICD9 code: 250), hyperlipidemia (ICD9 codes: 272), coronary artery disease (ICD9 codes: 410–414), cerebrovascular accident (430–438), chronic kidney disease (ICD9 code: 585), chronic obstructive pulmonary disease (COPD, ICD9 codes: 491, 492, 496), chronic liver disease (ICD9 code: 571.4), autoimmune diseases (including systemic lupus erythematosus, ICD9 code: 710.0; ankylosing spondylitis, ICD9 code: 720.0; rheumatoid arthritis, ICD-9-CM code: 714.0; Sjögren's syndrome, ICD-9-CM code: 710.2), psoriasis (ICD9 code: 696), vasculitis (ICD9 code: 446), and inflammatory bowel disease (IBD, including Crohn’s disease, ICD9 codes: 555.x and ulcerative colitis, ICD9 codes: 556.x).

### Statistical analysis

Chi-square test was used to determine the differences of baseline characteristics for categorical variables and Student’s *t*-test was applied to examine continuous variables between case group (bowel resection) and the control group. Standardized mean difference (SMD) was used to assess the difference between the case group and the control group after PS matching; a value of < 0.1 was considered negligible^13^. Incidence of NTS infection was estimated for both groups by the end of 2013. We used the Kaplan–Meier method to measure fractions free of NTS infection during the follow-up period in the two groups and used the log-rank test to examine the difference. Incidence rates (IRs) of NTS infection, per 10,000 person-years, were calculated for patients with or without bowel resections, gender, age group, occupation, and comorbidities. Cox proportional-hazards regression analysis was used to calculate the case group to the control group hazard ratio (HR) of NTS infection and 95% confidence interval (CI). Adjusted hazard ratio (aHR) was estimated after controlling for covariates. Gender-, age-, occupation-, and comorbidities-stratified analyses of NTS infection with or without bowel resection were conducted. All statistical analyses were performed using SAS software version 9.4 (SAS Institute INC., Carey, NC, USA). A two-tailed p-value below 0.05 was considered as significant.

### Sensitivity analysis

According to Wu et al.^14^, a temporal association between the use of proton pump inhibitors and increased susceptibility to NTS was demonstrated, which would bias our results. Therefore, we performed the sensitivity analysis by excluding peptic ulcer disease patients (ICD9 codes: 531, 532, 533, and 534) who had a high probability of taking proton pump inhibitors and PS matching.

## Results

### Demographic characteristics of patients

Our study population includes 35,851 cases and 35,851 controls. Table 1 shows that the male ratio and the mean age were 52% and 56.1 years in the case group, and 51% and 55.6 years in the control group, respectively. There was no significant difference in the baseline occupation and comorbidities. The overall median follow-up time was 4.53 years.

**Table 1:** Demographic Characteristics of Patients with and without Bowel Resection.

Variables	Bowel resection				SMD
	No (n = 35,851)		Yes (n = 35,851)		
	n	%	n	%	
**Gender**					0.01
Female	17,522	49%	17,300	48%	
Male	18,329	51%	18,551	52%	
**Age, year**					
18–30	3418	10%	3229	9%	0.02
31–40	4788	13%	4659	13%	0.01
41–50	6382	18%	6344	18%	0.003
> 50	21,263	59%	21,619	60%	0.02
Mean, (SD)	55.6	(18.5)	56.1	(18.3)	0.02
**Occupation**					
Officer	16,360	46%	16,721	47%	0.02
Worker	8198	23%	7964	22%	0.02
Farmer	685	2%	602	2%	0.02
Fisher	6215	17%	6275	18%	0.004
Others	4393	12%	4289	12%	0.01
**Comorbidities**					
Hypertension	10,584	30%	10,266	29%	0.02
Diabetes	5733	16%	5529	15%	0.02
Hyperlipidemia	2900	8%	2401	7%	0.05
Coronary artery disease	4129	12%	3830	11%	0.03
Cerebrovascular accident	3798	11%	3255	9%	0.05
Chronic kidney disease	1211	3%	1207	3%	0.001
COPD	2455	7%	2226	6%	0.03
Chronic liver disease	1836	5%	1631	5%	0.03
Autoimmune disease	451	1%	406	1%	0.01
Psoriasis	87	0.24%	74	0.21%	0.01
Vasculitis	27	0.08%	14	0.04%	0.02
IBD	673	2%	772	2%	0.02

*SD* standard deviation, *SMD* standardized mean difference, *COPD* chronic obstructive pulmonary disease, *IBD* Inflammatory bowel disease.

### Cumulative incidence and risk of NTS infection

**Figure 2:**
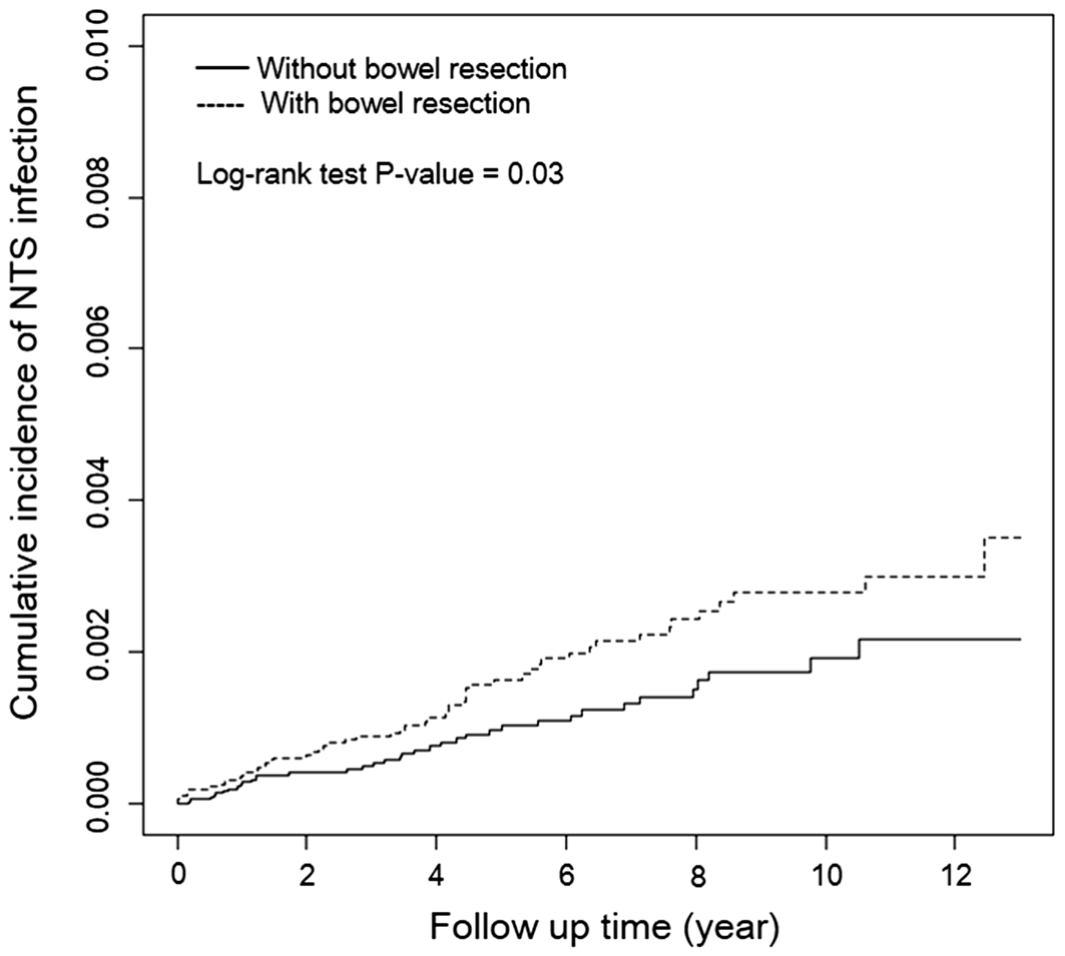
Cumulative incidence curves of nontyphoidal salmonella (NTS) infection among the propensity score–matched Cohort using The Kaplan–Meier method.

By the end of follow-up, the Kaplan–Meier analysis showed that the NTS infection was significantly more in the bowel resection group than in the control group (*p* = 0.03) (Fig. 2). The IR of NTS infection was higher in the bowel resection group than in the control group (2.97 vs. 1.92 per 10,000 person-years), with an aHR of 1.64 (95% CI = 1.08–2.48) for the bowel resection group (Table 2). Considering the location of bowel resection, the IR of NTS infection was higher in the small bowel resection group than in the control group (3.66 vs. 1.92 per 10,000 person-years), with an aHR of 1.96 (95% CI = 1.20–3.19) for the small bowel resection group, and higher in the right hemicolectomy group than in the control group (3.32 vs. 1.92 per 10,000 person-years), with an aHR of 1.96 (95% CI = 1.02–3.77) for the right hemicolectomy group. Besides, age (31–40 years, aHR of 4.71, 95% CI = 1.05–21.1; > 50, aHR of 5.25, 95% CI = 1.27–21.8), hypertension (aHR of 1.61, 95% CI = 1.00–2.58), chronic kidney disease (aHR of 2.35, 95% CI = 1.06–5.21), COPD (aHR of 2.18, 95% CI = 1.18–4.02), and autoimmune disease (aHR of 3.83, 95% CI = 1.4–10.47) also increased the risk of NTS infection significantly. Large bowel resection, left hemicolectomy, sigmoidectomy, total colectomy, and bowel anastomosis did not increase the NTS infection risk prominently.

**Table 2:** Incidence Rate and Hazard Ratio of NTS Infection.

Variables	NTS infection			cHR	(95% CI)	aHR†	(95% CI)
	n	PY	IR				
**Bowel resection**							
No	37	192,902	1.92	1.00	–	1.00	–
Yes	56	188,270	2.97	1.56	(1.03, 2.37)*	1.64	(1.08, 2.48)*
**Small bowel resection**							
No	37	192,902	1.92	1.00	–	1.00	–
Yes	29	79,213	3.66	1.93	(1.18, 3.14)**	1.96	(1.20, 3.19)**
**Large bowel resection**							
No	37	192,902	1.92	1.00	–	1.00	–
Yes	28	98,288	2.85	1.52	(0.93, 2.48)	1.59	(0.97, 2.60)
**Right hemicolectomy**							
No	37	192,902	1.92	1.00	–	1.00	–
Yes	12	36,091	3.32	1.77	(0.92, 3.39)	1.96	(1.02, 3.77)*
**Transverse colon resection**							
No	37	192,902	1.92	1.00	–		
Yes	0	2847	0.00	0.00			
**Left hemicolectomy**							
No	37	192,902	1.92	1.00	–	1.00	–
Yes	6	12,670	4.74	2.51	(1.06, 5.95)*	2.12	(0.89, 5.04)
**Sigmoidectomy**							
No	37	192,902	1.92	1.00	–	1.00	–
Yes	6	16,997	3.53	1.84	(0.78, 4.37)	1.66	(0.70, 3.96)
**Total colectomy**							
No	37	192,902	1.92	1.00	–		
Yes	0	3392	0.00	0.00			
**Bowel anastomosis**							
No	37	192,902	1.92	1.00	–	1.00	–
Yes	11	57,857	1.90	1.01	(0.52, 1.99)	1.18	(0.60, 2.33)
**Gender**							
Female	34	174,426	1.95	1.00	–		
Male	59	206,746	2.85	1.48	(0.97, 2.26)		
**Age, year**							
18–30	2	43,760	0.46	1.00	–	1.00	–
31–40	12	53,443	2.25	4.86	(1.09, 21.7)*	4.71	(1.05, 21.1)*
41–50	6	70,720	0.85	1.83	(0.37, 9.07)	1.67	(0.34, 8.31)
> 50	73	213,249	3.42	7.36	(1.81, 30.0)**	5.25	(1.27, 21.8)*
**Occupation**							
Officer	38	174,857	2.17	1.00	–		
Worker	16	88,397	1.81	0.83	(0.47, 1.50)		
Farmer	1	7159	1.40	0.65	(0.09, 4.70)		
Fisher	21	65,542	3.20	1.48	(0.87, 2.51)		
Others	17	45,218	3.76	1.73	(0.98, 3.06)		
**Comorbidities**							
**Hypertension**							
No	53	292,983	1.81	1.00	–	1.00	–
Yes	40	88,190	4.54	2.48	(1.64, 3.75)***	1.61	(1.00, 2.58)*
**Diabetes**							
No	72	332,861	2.16	1.00	–	1.00	–
Yes	21	48,312	4.35	1.98	(1.22, 3.23)**	1.28	(0.76, 2.14)
**Hyperlipidemia**							
No	84	358,349	2.34	1.00	–		
Yes	9	22,823	3.94	1.66	(0.84, 3.31)		
**Coronary artery disease**							
No	79	347,841	2.27	1.00	–	1.00	–
Yes	14	33,332	4.20	1.82	(1.03, 3.21)*	0.92	(0.50, 1.70)
**Cerebrovascular accident**							
No	85	353,319	2.41	1.00	–		
Yes	8	27,854	2.87	1.16	(0.56, 2.41)		
**Chronic kidney disease**							
No	86	373,043	2.31	1.00	–	1.00	–
Yes	7	8130	8.61	3.62	(1.67, 7.85)**	2.35	(1.06, 5.21)*
**COPD**							
No	80	362,642	2.21	1.00	–	1.00	–
Yes	13	18,530	7.02	3.12	(1.73, 5.61)***	2.18	(1.18, 4.02)*
**Chronic liver disease**							
No	88	366,101	2.40	1.00	–		
Yes	5	15,071	3.32	1.35	(0.55, 3.34)		
**Autoimmune disease**							
No	89	377,224	2.36	1.00	–	1.00	–
Yes	4	3948	10.13	4.26	(1.56, 11.59)**	3.83	(1.4, 10.47)**
**Psoriasis**							
No	93	380,403	2.44	1.00	–		
Yes	0	770	0.00	0.00			
**Vasculitis**							
No	93	380,965	2.44	1.00	–		
Yes	0	207	0.00	0.00			
**IBD**							
No	91	372,731	2.44	1.00	–		
Yes	2	8442	2.37	0.98	(0.24, 3.97)		

*NTS* nontyphoidal salmonella, *COPD* chronic obstructive pulmonary disease, *IBD* Inflammatory bowel disease, *PY* person-year, *IR* incidence rate per 10,000 person-year, *cHR* crude hazard ratio, *aHR* adjusted hazard ratio.

^†^Adjusted by age, hypertension, diabetes, coronary artery disease, chronic kidney disease, COPD and rheumatoid autoimmune disease.

*p value < 0.05; **p value < 0.01; ***p value < 0.001.

### Stratification analysis of nontyphoidal salmonella infection

The stratification analysis (Table 3) revealed that in those without comorbidities, compared with patients without bowel resections, patients who received bowel resections had significantly increased risk of NTS infection for those without hypertension (aHR of 2.34, 95% CI = 1.3–4.22), diabetes (aHR of 2.11, 95% CI = 1.29–3.46), hyperlipidemia (aHR of 1.91, 95% CI = 1.22–2.98), coronary artery disease (aHR of 1.82, 95% CI = 1.15–2.88), cerebrovascular accident (aHR of 1.59, 95% CI = 1.03–2.46), chronic kidney disease (aHR of 1.56, 95% CI = 1.01–2.39), COPD (aHR of 1.85, 95% CI = 1.17–2.93), chronic liver disease (aHR of 1.62, 95% CI = 1.05–2.48), vasculitis (aHR of 1.64, 95% CI = 1.08–2.48), or IBD (aHR of 1.59, 95% CI = 1.04–2.42). The significant association between bowel resection and NTS infection was not found in each age group of all patients, different genders, different occupations, patients without autoimmune disease or psoriasis, and patients with comorbidities.

**Table 3:** The Stratification Analysis of NTS Infection.

Variables	Without bowel resection			With bowel resection			cHR	(95% CI)	aHR†	(95% CI)
	n	PY	IR	n	PY	IR				
**Gender**										
Female	14	88,765	1.58	20	85,661	2.33	1.51	(0.76, 3.00)	1.61	(0.81, 3.19)
Male	23	104,137	2.21	36	102,609	3.51	1.59	(0.94, 2.68)	1.63	(0.96, 2.75)
**Age, year**										
18–30	1	22,053	0.45	1	21,707	0.46	1.07	(0.07, 17.1)	1.71	(0.10, 30.2)
31–40	3	27,077	1.11	9	26,366	3.41	3.19	(0.86, 11.8)	3.66	(0.94, 14.2)
41–50	3	35,047	0.86	3	35,674	0.84	0.97	(0.19, 4.80)	1.07	(0.21, 5.34)
> 50	30	108,726	2.76	43	104,523	4.11	1.49	(0.93, 2.38)	1.54	(0.96, 2.45)
**Occupation**										
Officer	14	86,403	1.62	24	88,454	2.71	1.70	(0.88, 3.28)	1.77	(0.92, 3.43)
Worker	8	45,223	1.77	8	43,174	1.85	1.03	(0.38, 2.75)	1.07	(0.40, 2.88)
Farmer	0	3893	0.00	1	3266	3.06				
Fisher	9	33,987	2.65	12	31,554	3.80	1.44	(0.60, 3.41)	1.44	(0.61, 3.43)
Others	6	23,397	2.56	11	21,821	5.04	2.01	(0.74, 5.44)	2.02	(0.75, 5.47)
**Comorbidities**										
**Hypertension**										
No	16	145,886	1.10	37	147,097	2.52	2.31	(1.28, 4.15)**	2.34	(1.3, 4.22)**
Yes	21	47,017	4.47	19	41,173	4.61	1.04	(0.56, 1.93)	1.07	(0.58, 2.00)
**Diabetes**										
No	24	167,344	1.43	48	165,517	2.90	2.04	(1.25, 3.33)**	2.11	(1.29, 3.46)**
Yes	13	25,559	5.09	8	22,753	3.52	0.70	(0.29, 1.68)	0.74	(0.30, 1.78)
**Hyperlipidemia**										
No	30	179,471	1.67	54	178,879	3.02	1.82	(1.16, 2.84)**	1.91	(1.22, 2.98)**
Yes	7	13,432	5.21	2	9392	2.13	0.41	(0.09, 2.00)	0.37	(0.08, 1.78)
**Coronary artery disease**										
No	29	174,143	1.67	50	173,698	2.88	1.74	(1.10, 2.75)*	1.82	(1.15, 2.88)*
Yes	8	18,759	4.26	6	14,572	4.12	0.96	(0.33, 2.76)	0.90	(0.31, 2.58)
**Cerebrovascular accident**										
No	34	176,656	1.92	51	176,663	2.89	1.51	(0.98, 2.33)	1.59	(1.03, 2.46)*
Yes	3	16,247	1.85	5	11,607	4.31	2.34	(0.56, 9.79)	2.21	(0.53, 9.27)
**Chronic kidney disease**										
No	35	188,352	1.86	51	184,691	2.76	1.50	(0.97, 2.30)	1.56	(1.01, 2.39)*
Yes	2	4551	4.39	5	3579	13.97	3.19	(0.62, 16.5)	2.63	(0.50, 13.8)
**COPD**										
No	29	182,097	1.59	51	180,545	2.82	1.78	(1.13, 2.82)*	1.85	(1.17, 2.93)**
Yes	8	10,806	7.40	5	7725	6.47	0.86	(0.28, 2.62)	0.87	(0.28, 2.70)
**Chronic liver disease**										
No	35	184,434	1.90	53	181,667	2.92	1.55	(1.01, 2.37)*	1.62	(1.05, 2.48)*
Yes	2	8468	2.36	3	6603	4.54	1.95	(0.33, 11.7)	1.88	(0.31, 11.3)
**Autoimmune disease**										
No	37	190,628	1.94	52	186,596	2.79	1.45	(0.95, 2.21)	1.51	(0.99, 2.31)
Yes	0	2274	0.00	4	1674	23.89				
**Psoriasis**										
No	37	192,392	1.92	56	188,011	2.98	1.56	(1.03, 2.37)*	1.51	(0.99, 2.31)
Yes	0	510	0.00	0	259	0.00				
**Vasculitis**										
No	37	192,762	1.92	56	188,204	2.98	1.56	(1.03, 2.37)*	1.64	(1.08, 2.48)*
Yes	0	141	0.00	0	67	0.00				
**IBD**										
No	37	188,948	1.96	54	183,783	2.94	1.52	(1.00, 2.3)	1.59	(1.04, 2.42)*
Yes	0	3955	0.00	2	4487	4.46				

*NTS* nontyphoidal salmonella, *COPD* chronic obstructive pulmonary disease, *IBD* inflammatory bowel disease, *PY* person-year, *IR* incidence rate per 10,000 person-year, *cHR* crude hazard ratio, *aHR* adjusted hazard ratio.

^†^Adjusted by age, hypertension, diabetes, coronary artery disease, chronic kidney disease, COPD and rheumatoid autoimmune disease.

*p value < 0.05, **p value < 0.01, ***p value < 0.001.

### Results of sensitivity analysis

Results of our sensitivity analysis were almost consistent with those of our primary analyses (Table 4). The differences are that our sensitivity analysis even indicated that patients would have an increased risk of NTS infection receiving large bowel resection (aHR of 1.69, 95% CI = 1.02–2.80) or left hemicolectomy (aHR of 2.41, 95% CI = 1.01–5.77). The patient baseline characteristics in our sensitivity analysis are shown in Table 5.

**Table 4:** Sensitivity Analysis (Excluding Peptic Ulcer Disease Patients).

Type of bowel resection	NTS infection	cHR	(95% CI)	aHR†	(95% CI)
	n				
Bowel resection	52	1.58	(1.02, 2.43)*	1.66	(1.07, 2.56)*
Small bowel resection	27	1.93	(1.17, 3.21)*	1.92	(1.04, 3.19)*
Large bowel resection	27	1.58	(0.95, 2.62)	1.69	(1.02, 2.80)*
Right hemicolectomy	12	1.90	(0.98, 3.68)	2.27	(1.17, 4.43)*
Transverse colon resection	0				
Left hemicolectomy	6	2.71	(1.14, 6.46)*	2.41	(1.01, 5.77)*
Sigmoidectomy	6	2.01	(0.84, 4.78)	1.91	(0.79, 4.57)
Total colectomy	0				
Bowel anastomosis	9	0.90	(0.43, 1.88)	1.06	(0.51, 2.23)

*NTS* nontyphoidal salmonella, *n* number of event, *cHR* crude hazard ratio, *aHR* adjusted hazard ratio.

^†^Adjusted by age, hypertension, diabetes, coronary artery disease, chronic kidney disease, COPD and rheumatoid autoimmune disease.

*p value < 0.05, **p value < 0.01, ***p value < 0.001.

**Table 5:** Demographic Characteristics of Patients with and without Bowel Resection (Excluding Peptic Ulcer Disease Patients).

Variables	Bowel resection				SMD
	No (n = 35,004)		Yes (n = 35,004)		
	n	%	n	%	
**Gender**					0.01
Female	17,098	49%	16,886	48%	
Male	17,906	51%	18,118	52%	
**Age, year**					
18–30	3396	10%	3195	9%	0.02
31–40	4621	13%	4592	13%	0.002
41–50	6345	18%	6217	18%	0.01
> 50	20,642	59%	21,000	60%	0.02
Mean, (SD)	55.4	(18.4)	56.0	(18.3)	0.03
**Occupation**					
Officer	16,298	47%	16,332	47%	0.002
Worker	7586	22%	7762	22%	0.01
Farmer	738	2%	582	2%	0.03
Fisher	6076	17%	6119	17%	0.00
Others	4306	12%	4209	12%	0.01
**Comorbidities**					
Hypertension	10,099	29%	9965	28%	0.01
Diabetes	6247	18%	5371	15%	0.07
Hyperlipidemia	2683	8%	2329	7%	0.04
Coronary artery disease	4192	12%	3714	11%	0.04
Cerebrovascular accident	3674	10%	3159	9%	0.05
Chronic kidney disease	1111	3%	1176	3%	0.01
COPD	2449	7%	2153	6%	0.03
Chronic liver disease	1977	6%	1585	5%	0.05
Autoimmune disease	400	1%	390	1%	0.003
Psoriasis	72	0.2%	74	0.2%	0.001
Vasculitis	166	0.5%	14	0.04%	0.09
IBD	665	2%	752	2%	0.02

*SD* standard deviation, *SMD* standardized mean difference, *COPD* chronic obstructive pulmonary disease, *IBD* inflammatory bowel disease.

## Discussion

Our study first demonstrated that bowel resection was associated with a significantly increased risk of NTS infection. Of note, the HR of NTS infection for patients with small bowel resection or right hemicolectomy was nearly two times greater than for those without small bowel resection or right hemicolectomy.

We hypothesized that based on the bowel resection site, the microbiota composition change would result in NTS infection. As mentioned above, the reported bacterial species in the intestine included Bacilli (Firmicutes), Streptococcaceae (Firmicutes), Actinobacteria, Clostridium, Staphylococcus, Bacteroides, Lactobacillus, Enterococcus, Bifidobacterium, Enterobacteriaceae, and Ruminococcus. Oh et al. demonstrated the potential protective effects of using *Bacillus subtilis* CSL2, against *Salmonella* *gallinarum* infection on laying hens^15^. Actinomyces might have antimicrobial potency toward NTS ^16^. Jacobson et al. discovered that in mice, *Bacteroides* spp. limit intestinal *Salmonella typhimurium* expansion and fecal shedding^17^. Lactobacillus has remarkable anti-salmonella activities in vivo and in vitro^18,19^. Strains of *Bifidobacterium* were antagonistic to *Salmonella*^20^. *Bifidobacterium thermophilum* RBL67 influences the transcriptome of *Salmonella* and causes an imbalanced virulence gene expression, but can protect hosts from infection^21^. *Clostridia*, including the genera *Roseburia* and *Blautia* from the family *Lachnospiraceae* and the genera *Ruminococcus* and *Anaerovibrio*, were more abundant in *Salmonella*-negative pigs^8^. A metabolite from bacteria—indole, is probably associated with NTS infection in our gut. Indole can prevent NTS infection because of its effects on decreasing *Salmonella* invasion in vivo, *Salmonella* motility, virulence gene expression, and increasing epithelial cells’ resistance to *Salmonella* invasion^22^.

Gordon et al. indicated that host risk factors included extremes of age and immunocompromised status such as rheumatologic disease, which corresponded with our results^3^. We discovered that patients aged 31–40 years had an increased risk of NTS infection, which was first reported by us. Our study demonstrated other risk factors for NTS infection like hypertension, chronic kidney disease, and COPD. Atherosclerosis was demonstrated a risk factor for endovascular *Salmonella* bacteremia^23^. Traditional risk factors for atherosclerosis included hypertension, so the hypertension might be associated with NTS infection^24^. Chronic kidney disease was not identified as a risk factor for NTS infection before. The possible explanation is that chronic kidney disease could result in immunocompromised status so that patients easily got NTS infection. Lisowska’s study showed peripheral CD4 + and CD8 + T cells and B cells in the blood decrease^25^, and Fernández-Fresnedo et al. indicated an increased incidence of apoptosis in B cells in chronic kidney disease patients^26^. In end-stage renal disease patients, increased apoptosis and diminished populations of naïve and central memory T cells were found, and their antigen-specific memory CD4 + T cells were impaired^27^. In smokers with COPD, Knobloch’s research revealed that interferon-γ release from ex vivo generated CD4^+^ effector cells of the Th1 subtype upon challenge with lipopolysaccharide (LPS) purified from *Salmonella minnesota* would decrease^28^.

Our stratification analysis showed patients without hypertension, diabetes, hyperlipidemia, coronary artery disease, cerebrovascular accident, chronic kidney disease, COPD, chronic liver diseases, vasculitis, or IBD would be at higher risk of NTS infection after bowel resection. We might explain this phenomenon by the fact that affected patients usually have underlying diseases, so that NTS poses significant health threats^4^. If underlying covariates did not exist, bowel resection would play a significant predisposing factor of NTS infection. Our results reaffirm that in patients without underlying diseases, bowel resection should be considered carefully to avoid possible NTS infection in the future.

Our study has the advantage that it is a large-scale nationwide analysis with longitudinal follow-up. However, we admit several limitations to our study. First, we lacked information regarding body mass index, alcohol consumption, betel nut use, smoking, and other immunomodulator prescription. However, we used adjusted covariates, such as autoimmune diseases, psoriasis, and IBD, which could represent immune-related medication use. Second, the potential misclassification in establishing study cohorts might exist. We used bowel resection procedure codes to identify the case group. The policy of patient anonymity within the NHIRD prevented us from confirming patients’ diagnoses. Third, we demonstrated that right hemicolectomy in our primary analyses and left hemicolectomy in our sensitivity analysis increased the risk of NTS infection, but we did not discover any reference which could explain the mechanism.

## Conclusions

Bowel resection causes an increased risk of NTS infection. Patients aged 31–40 and > 50 years or those with comorbidities such as hypertension, chronic kidney disease, COPD, and autoimmune disease have an elevated risk of NTS infection. Furthermore, in patients without comorbidities, bowel resection should be considered carefully.
